# Supplementary material for: Analyses of child cardiometabolic phenotype following assisted reproductive technologies using a pragmatic trial emulation approach
Source: Nat Commun. 2021 Sep 23;12:5613. doi: 10.1038/s41467-021-25899-4 (PMC8460697; doi:10.1038/s41467-021-25899-4)
Supplement: Supplementary file 1 — Supplementary Information [file 41467_2021_25899_MOESM1_ESM.pdf]

TITLE

Analyses of child cardiometabolic phenotype following assisted reproductive technologies using a pragmatic trial emulation approach.

SUPPLEMENTAL TABLES

Supplemental Table 1. Target pragmatic trial amongst possible subfertile couples, by conception status.

Supplemental Table 2. Child characteristics and anthropometrics, by conception status subgroups.

Supplemental Table 3. Child fat and lean mass at 5 and 6 years and serum cardiometabolic biomarkers at 6 years, adjusted for pre-pregnancy and pregnancy factors.

Supplemental Table 4. Child illnesses reported at 12-month visit, in the past three months.

SUPPLEMENTAL FIGURES

Supplemental Figure 1. Difference in ultrasound-estimated weight of ART-conceived foetuses versus spontaneously-conceived foetuses, compared to the subfertile cohort (A) and adjusted for pre-pregnancy characteristics (B).

Supplemental Figure 2. QMR-measured body composition at years 5 (A) and 6 (B) and distributions of serum cardiometabolic biomarkers and blood pressure at 6 years (C), by ART status.

Supplemental Figure 3. Difference in anthropometrics (A), skinfold thickness (B), and blood pressures (C) comparing ART- and spontaneously-conceived foetuses, estimated by collaborative-targeted maximum likelihood estimation (C-TMLE).

Supplemental Figure 4. Length-for-age Z-score and ART status over time, by comparison cohort.

Supplemental Figure 5. Associations between ART status and anthropometry, accounting for differential follow-up at each study visit.

Supplemental Figure 6. EWAS of ART status.

Supplemental Figure 7. Negative control analyses of mediation by cg03904042 (A) and cg27146050 (B) methylation in maternal mid-pregnancy peripheral blood, target trial subcohort.

Supplemental Figure 8. Subject retention (A) and distribution of observed heights (B), by ART and eventual loss-to-follow-up status.

Supplemental Figure 9. Predicted average anthropometrics by ART status, under a linear mixed effects model with individual-level random intercepts and slopes.

Supplemental Figure 10. Negative Control Mediation by Maternal DNA methylation (DNAm).

Supplemental Table 1. Target pragmatic trial amongst possible subfertile couples, by conception status.			
	**Spontaneous, Possibly subfertile (n=93)**	**ART (n=83)**	**Overall (n=176)**
**Mother's Age at Delivery (Years)**			
Mean (SD)	33.5 (± 4.67)	34.5 (± 2.97)	34.0 (± 3.98)
**Nulliparous**			
Yes	38 (40.9%)	67 (80.7%)	105 (59.7%)
No	55 (59.1%)	16 (19.3%)	71 (40.3%)
**Mother's Ethnicity**			
Chinese	67 (72.0%)	64 (77.1%)	131 (74.4%)
Malay	12 (12.9%)	8 (9.6%)	20 (11.4%)
Indian	14 (15.1%)	11 (13.3%)	25 (14.2%)
Other	0 (0%)	0 (0%)	0 (0%)
**Mother's Highest Educational Qualification**			
No Education	0 (0%)	0 (0%)	0 (0%)
Primary	5 (5.4%)	0 (0%)	5 (2.8%)
Secondary	14 (15.1%)	13 (15.7%)	27 (15.3%)
ITE / NTC	1 (1.1%)	4 (4.8%)	5 (2.8%)
GCE A-Level	21 (22.6%)	24 (28.9%)	45 (25.6%)
University	52 (55.9%)	41 (49.4%)	93 (52.8%)
Missing	0 (0%)	1 (1.2%)	1 (0.6%)
**Monthly Household Income (SGD)**			
0 to 999	1 (1.1%)	0 (0%)	1 (0.6%)
1000 to 1999	8 (8.6%)	4 (4.8%)	12 (6.8%)
2000 to 3999	17 (18.3%)	12 (14.5%)	29 (16.5%)
4000 to 5999	27 (29.0%)	18 (21.7%)	45 (25.6%)
6000+	38 (40.9%)	41 (49.4%)	79 (44.9%)
Missing	2 (2.2%)	8 (9.6%)	10 (5.7%)
**Mother's Height (cm)**			
Mean (SD)	159 (± 5.64)	159 (± 5.56)	159 (± 5.60)
Missing	1 (1.1%)	2 (2.4%)	3 (1.7%)
**Mother's pre-pregnancy BMI (kg/m^2)**			
Mean (SD)	22.4 (± 3.73)	23.4 (± 4.78)	22.8 (± 4.26)
Missing	5 (5.4%)	8 (9.6%)	13 (7.4%)
**Maternal Fasting Glucose (mmol/L)**			
Mean (SD)	4.34 (± 0.408)	4.48 (± 0.642)	4.41 (± 0.532)
Missing	3 (3.2%)	5 (6.0%)	8 (4.5%)
**Maternal 2-hour post-OGTT Glucose (mmol/L)**			
Mean (SD)	6.85 (± 1.45)	7.21 (± 1.75)	7.02 (± 1.60)
Missing	3 (3.2%)	5 (6.0%)	8 (4.5%)
**Maternal High Blood Pressure History**			
No	66 (71.0%)	60 (72.3%)	126 (71.6%)
Yes	7 (7.5%)	11 (13.3%)	18 (10.2%)
Missing	20 (21.5%)	12 (14.5%)	32 (18.2%)

Supplemental Table 1. Target pragmatic trial amongst possible subfertile couples, by conception status.			
	**Spontaneous, Possibly subfertile (n=93)**	**ART (n=83)**	**Overall (n=176)**
**Any Smoking in Home (During Pregnancy)**			
No	72 (77.4%)	64 (77.1%)	136 (77.3%)
Yes	17 (18.3%)	15 (18.1%)	32 (18.2%)
Missing	4 (4.3%)	4 (4.8%)	8 (4.5%)
**Father's Age at Delivery (Years)**			
Mean (SD)	35.7 (± 5.07)	39.1 (± 4.64)	37.3 (± 5.14)
Missing	25 (26.9%)	26 (31.3%)	51 (29.0%)
**Father's Height (cm)**			
Mean (SD)	172 (± 5.48)	172 (± 5.96)	172 (± 5.68)
Missing	32 (34.4%)	31 (37.3%)	63 (35.8%)
**Father's Weight (kg)**			
Mean (SD)	77.6 (± 13.3)	76.0 (± 15.5)	76.9 (± 14.3)
Missing	32 (34.4%)	31 (37.3%)	63 (35.8%)
**Paternal Diabetes**			
No	69 (74.2%)	53 (63.9%)	122 (69.3%)
Yes	1 (1.1%)	4 (4.8%)	5 (2.8%)
Missing	23 (24.7%)	26 (31.3%)	49 (27.8%)
**Paternal High Blood Pressure History**			
No	60 (64.5%)	45 (54.2%)	105 (59.7%)
Yes	10 (10.8%)	12 (14.5%)	22 (12.5%)
Missing	23 (24.7%)	26 (31.3%)	49 (27.8%)

Supplemental Table 2. Child characteristics and anthropometrics, by conception status subgroups.					
	**ART (n=83)**	**Spontaneous (fertility indications only) (n=93)**	**Spontaneous (maternal + paternal risk factors) (n=200)**	**Spontaneous (paternal risk factors only) (n=121)**	**Spontaneous (no indications, 20% random sample) (n=204)**
**Child Sex**					
Female	35 (42.2%)	50 (53.8%)	96 (48.0%)	54 (44.6%)	98 (48.0%)
Male	48 (57.8%)	43 (46.2%)	104 (52.0%)	67 (55.4%)	106 (52.0%)
**Gestational Age @ Birth (weeks)**					
Mean (SD)	38.3 (± 1.84)	38.9 (± 1.71)	38.8 (± 1.61)	38.8 (± 1.48)	39.0 (± 1.19)
**Mode of Delivery**					
Caesarean	17 (20.5%)	28 (30.1%)	47 (23.5%)	22 (18.2%)	34 (16.7%)
Vaginal	66 (79.5%)	65 (69.9%)	153 (76.5%)	99 (81.8%)	170 (83.3%)
**Birth Weight (kg)**					
Mean (SD)	3.06 (± 0.483)	3.10 (± 0.461)	3.11 (± 0.457)	3.11 (± 0.452)	3.14 (± 0.419)
**Birth Length (cm)**					
Mean (SD)	48.0 (± 2.68)	48.9 (± 2.25)	48.9 (± 2.27)	48.9 (± 2.20)	48.8 (± 2.28)
**Months Exclusively Breastfed**					
< 1	60 (72.3%)	65 (69.9%)	153 (76.5%)	99 (81.8%)	142 (69.6%)
1 to <3	1 (1.2%)	1 (1.1%)	4 (2.0%)	3 (2.5%)	18 (8.8%)
3 to <6	6 (7.2%)	10 (10.8%)	19 (9.5%)	10 (8.3%)	20 (9.8%)
6 to 12	6 (7.2%)	11 (11.8%)	18 (9.0%)	9 (7.4%)	9 (4.4%)
12+	0 (0%)	0 (0%)	0 (0%)	0 (0%)	0 (0%)
**Weight @ 1 yr (kg)**					
Mean (SD)	9.35 (± 1.04)	9.48 (± 1.03)	9.41 (± 1.03)	9.33 (± 1.04)	9.41 (± 1.09)
Missing	15 (18.1%)	14 (15.1%)	22 (11.0%)	9 (7.4%)	41 (20.1%)
**Weight @ 2 yr (kg)**					
Mean (SD)	11.6 (± 1.27)	12.0 (± 1.34)	12.0 (± 1.35)	12.0 (± 1.37)	12.2 (± 1.66)
Missing	21 (25.3%)	12 (12.9%)	15 (7.5%)	3 (2.5%)	44 (21.6%)
**Weight @ 5 yr (kg)**					
Mean (SD)	18.0 (± 4.00)	18.8 (± 3.20)	18.8 (± 3.27)	18.8 (± 3.46)	18.9 (± 3.52)
Missing	22 (26.5%)	13 (14.0%)	30 (15.0%)	19 (15.7%)	55 (27.0%)
**Weight @ 6 yr (kg)**					
Mean (SD)	19.7 (± 3.49)	21.3 (± 4.13)	21.3 (± 4.27)	21.3 (± 4.50)	21.0 (± 3.68)
Missing	24 (28.9%)	11 (11.8%)	32 (16.0%)	21 (17.4%)	60 (29.4%)
**Length @ 1 yr (cm)**					
Mean (SD)	75.1 (± 2.61)	75.6 (± 3.42)	75.8 (± 3.15)	75.9 (± 2.93)	75.5 (± 3.19)
Missing	15 (18.1%)	14 (15.1%)	22 (11.0%)	9 (7.4%)	41 (20.1%)
**Height @ 2 yr (cm)**					
Mean (SD)	86.9 (± 3.07)	87.7 (± 3.36)	88.0 (± 3.56)	88.1 (± 3.67)	87.9 (± 3.81)
Missing	23 (27.7%)	13 (14.0%)	20 (10.0%)	7 (5.8%)	52 (25.5%)
**Height @ 3 yr (cm)**					
Mean (SD)	94.1 (± 3.66)	95.3 (± 3.56)	95.3 (± 3.63)	95.4 (± 3.76)	95.1 (± 3.99)

Supplemental Table 2. Child characteristics and anthropometrics, by conception status subgroups.					
	ART (n=83)	Spontaneous (fertility indications only) (n=93)	Spontaneous (maternal + paternal risk factors) (n=200)	Spontaneous (paternal risk factors only) (n=121)	Spontaneous (no indications, 20% random sample) (n=204)
Missing	19 (22.9%)	9 (9.7%)	15 (7.5%)	6 (5.0%)	49 (24.0%)
**Height @ 4 yr (cm)**					
Mean (SD)	102 (± 3.85)	103 (± 3.96)	103 (± 4.07)	103 (± 4.14)	102 (± 4.36)
Missing	26 (31.3%)	9 (9.7%)	23 (11.5%)	15 (12.4%)	54 (26.5%)
**Height @ 5 yr (cm)**					
Mean (SD)	108 (± 4.41)	110 (± 4.48)	110 (± 4.38)	110 (± 4.44)	110 (± 4.61)
Missing	22 (26.5%)	13 (14.0%)	30 (15.0%)	19 (15.7%)	55 (27.0%)
**Height @ 6 yr (cm)**					
Mean (SD)	114 (± 4.91)	116 (± 4.83)	116 (± 4.73)	116 (± 4.72)	116 (± 5.18)
Missing	24 (28.9%)	11 (11.8%)	32 (16.0%)	21 (17.4%)	60 (29.4%)
**Fasting Glucose @ 6 yr (mmol/L)**					
Mean (SD)	4.60 (± 0.380)	4.47 (± 0.483)	4.50 (± 0.448)	4.54 (± 0.410)	4.55 (± 0.336)
Missing	49 (59.0%)	43 (46.2%)	92 (46.0%)	53 (43.8%)	110 (53.9%)
**Systolic BP @ 6 yr (mmHg)**					
Mean (SD)	97.5 (± 8.26)	102 (± 9.26)	102 (± 8.51)	102 (± 8.05)	100 (± 6.98)
Missing	36 (43.4%)	27 (29.0%)	58 (29.0%)	35 (28.9%)	83 (40.7%)
**Diastolic BP @ 6 yr (mmHg)**					
Mean (SD)	57.3 (± 4.99)	59.2 (± 5.85)	59.3 (± 5.68)	59.8 (± 6.01)	59.4 (± 5.20)
Missing	36 (43.4%)	27 (29.0%)	58 (29.0%)	35 (28.9%)	83 (40.7%)

Supplemental Table 3. Child body composition at 5 and 6 years and serum cardiometabolic biomarkers at 6 years, adjusted for pre-pregnancy and pregnancy factors.

	Crude<br>(β [95% CI])	Adjusted for pre-pregnancy factors[1]<br>(β [95% CI])	Adjusted for pre-pregnancy + pregnancy factors[2]<br>(β [95% CI])
**QMR body composition at 5 years (N = 247)**			
fat mass (kg)	-0.9 (-1.8, -0.04)*	-0.7 (-1.6, 0.2)	-0.6 (-1.6, 0.3)
lean mass (kg)	-0.2 (-1.1, 0.6)	-0.2 (-1.0, 0.6)	-0.1 (-0.9, 0.7)
**QMR body composition at 6 years (N = 379)**			
fat mass (kg)	-0.8 (-1.9, 0.2)	-0.8 (-1.9, 0.2)	-0.8 (-1.8, 0.3)
lean mass (kg)	0.1 (-0.8, 0.9)	-0.4 (-1.2, 0.5)	-0.3 (-1.1, 0.6)
**Serum cardiometabolic biomarkers at 6 years**			
Creatinine (umol/L; N = 410)	-0.6 (-2.5, 1.3)	-0.3 (-2.3, 1.7)	-0.3 (-2.4, 1.8)
ALT (U/L; N = 400)	-0.9 (-2.6, 0.9)	-1.3 (-3.1, 0.4)	-1.4 (-3.3, 0.4)
AST (U/L; N = 407)	-0.9 (-3.2, 1.3)	-0.5 (-2.9, 1.9)	-0.4 (-2.9, 2.2)
log(GGT) (U/L; N = 337)	-0.03 (-0.1, 0.1)	-0.05 (-0.2, 0.1)	-0.06 (-0.2, 0.05)
HDL (mmol/L; N = 411)	0.07 (-0.03, 0.2)	0.06 (-0.04, 0.2)	0.07 (-0.03, 0.2)
LDL (mmol/L; N = 410)	0.2 (-0.1, 0.4)	0.2 (-0.05, 0.5)	0.3 (-0.02, 0.5)
log(Triglycerides) (mmol/L; N = 412)	-0.08 (-0.2, 0.1)	0.3 (-0.1, 0.7)	0.4 (-0.03, 0.7)
Total cholesterol (mmol/L; N = 412)	0.2 (-0.2, 0.5)	-0.06 (-0.2, 0.1)	-0.04 (-0.2, 0.1)
Cholesterol / HDL Ratio (N = 411)	-0.03 (-0.3, 0.2)	0.05 (-0.2, 0.3)	0.08 (-0.2, 0.3)
Fasting glucose (nmol/L; N = 524)	0.06 (-0.1, 0.2)	0.03 (-0.1, 0.2)	0.03 (-0.1, 0.2)
Fasting insulin (mU/L; N = 412)	-0.9 (-2.1, 0.3)	-0.8 (-2.2, 0.4)	-1.1 (-2.5, 0.2)
HOMA-β (%; N = 405)	-24 (-51, 3)	-22 (-50, 6)	-30 (-59, -0.2)*
HOMA-IR (N = 412)	-0.2 (-0.5, 0.1)	-0.2 (-0.5, 0.1)	-0.2 (-0.5, 0.1)
log(hs-CRP) (mg/L; N = 365)	-0.08 (-0.6, 0.4)	-0.2 (-0.7, 0.3)	-0.2 (-0.8, 0.3)

[1] Multivariable linear regression adjusted for: maternal age, education, ethnicity, household income, height, pre-pregnancy BMI, parity, and any tobacco smoke exposure in pregnancy; paternal height and weight; child sex, and polygenic risk score for adult adiposity. Multiple imputation by chained equations were used to estimate associations while simultaneously accounting for missing covariate values.
[2] Adjusted for variables in (1) and additionally for: maternal fasting and 2-hours post oral glucose tolerance test at 26 weeks of gestation; any resting blood pressure measurements exceeding 140 mmHg systolic or 90 mmHg diastolic at any time during pregnancy; gestational age at delivery; exact age of child at visit in days.

Supplemental Table 4. Child illnesses reported at 12-month visit, in the past three months.			
	**ART (n=68)**	**Spontaneous Conception (n=899)**	**Overall (n=967)**
**Diarrhea lasting 2+ days**			
No	57 (83.8%)	750 (83.4%)	807 (83.5%)
Yes	10 (14.7%)	148 (16.5%)	158 (16.3%)
Missing	1 (1.5%)	1 (0.1%)	2 (0.2%)
**Any fevers > 38 degrees C**			
No	43 (63.2%)	550 (61.2%)	593 (61.3%)
Yes	24 (35.3%)	344 (38.3%)	368 (38.1%)
Missing	1 (1.5%)	5 (0.6%)	6 (0.6%)
**Average # fevers (among any fevers)**			
Mean (SD)	1.3 (± 0.70)	1.3 (± 0.88)	1.3 (± 0.87)
Missing	44 (64.7%)	553 (61.5%)	597 (61.7%)
**Any hospital admissions**			
No	66 (97.1%)	867 (96.4%)	933 (96.5%)
Yes	1 (1.5%)	30 (3.3%)	31 (3.2%)
Missing	1 (1.5%)	2 (0.2%)	3 (0.3%)
**Any antibiotics use**			
No	55 (80.9%)	706 (78.5%)	761 (78.7%)
Yes	12 (17.6%)	189 (21.0%)	201 (20.8%)
Missing	1 (1.5%)	4 (0.4%)	5 (0.5%)
**Average # days of antibiotics (among users)**			
Mean (SD)	6.1 (± 3.3)	6.5 (± 4.8)	6.4 (± 4.7)
Missing	56 (82.4%)	720 (80.1%)	776 (80.2%)
**Any other diagnoses**			
No	42 (61.8%)	599 (66.6%)	641 (66.3%)
Yes	25 (36.8%)	293 (32.6%)	318 (32.9%)
Missing	1 (1.5%)	7 (0.8%)	8 (0.8%)
**Average # other diagnoses (among any diagnoses)**			
Mean (SD)	1.7 (± 0.94)	1.4 (± 0.72)	1.5 (± 0.74)
Missing	43 (63.2%)	601 (66.9%)	644 (66.6%)

SUPPLEMENTAL FIGURES

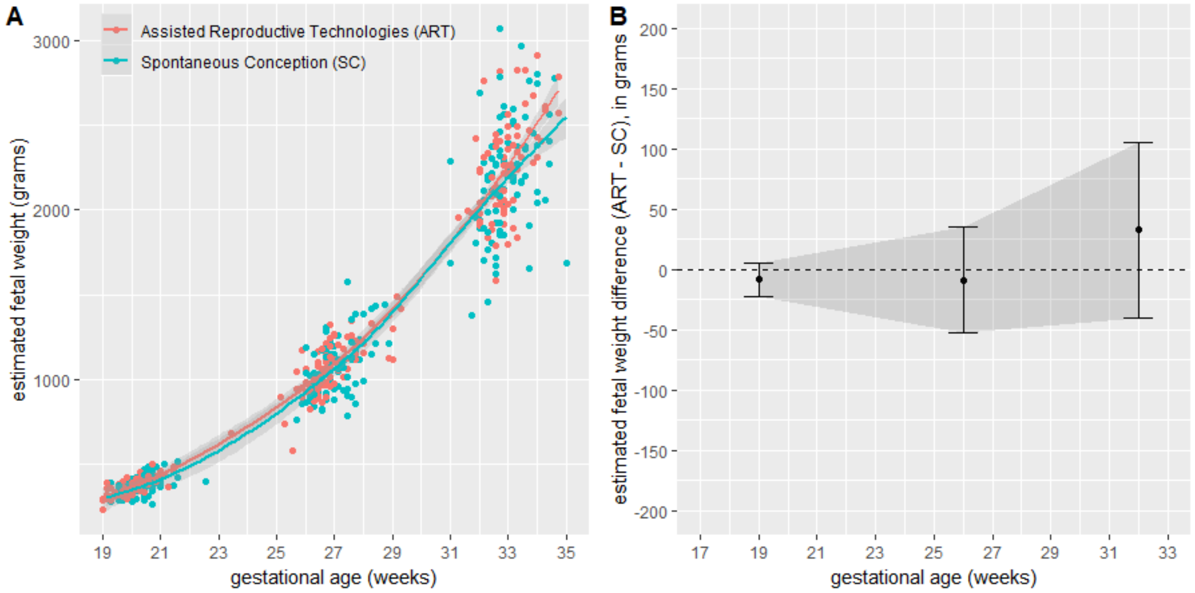

Supplemental Figure 1. **Difference in ultrasound-estimated weight of ART-conceived foetuses (N = 83) versus spontaneously-conceived foetuses (N = 93), compared to the subfertile cohort (A) and adjusted for pre-pregnancy characteristics (B).** (A) raw distribution and LOESS smoother-estimated 95% confidence shaded regions (B) point estimates and Wald-type 95% confidence intervals shown for multivariable linear regressions adjusted for maternal age, education, ethnicity, household income, height, pre-pregnancy BMI, parity, and any tobacco exposure in the home; paternal height and weight; child size, and polygenic risk score for adult adiposity with no multiple testing adjustments. Multiple imputation by chained equations were used to estimate associations while simultaneously accounting for missing covariate values. Shaded regions provide no additional information. The subfertile cohort was selected on the basis of medical history that may prompt couples to seek fertility specialist care including: history of 2 or more past miscarriages; medications with potential fertility indications (*e.g.* aspirin, hormones, thyroid, weight loss); history of PCOS, endometriosis, ovarian cysts, fibroids, or thyroid disorders (hyper- or hypo-).

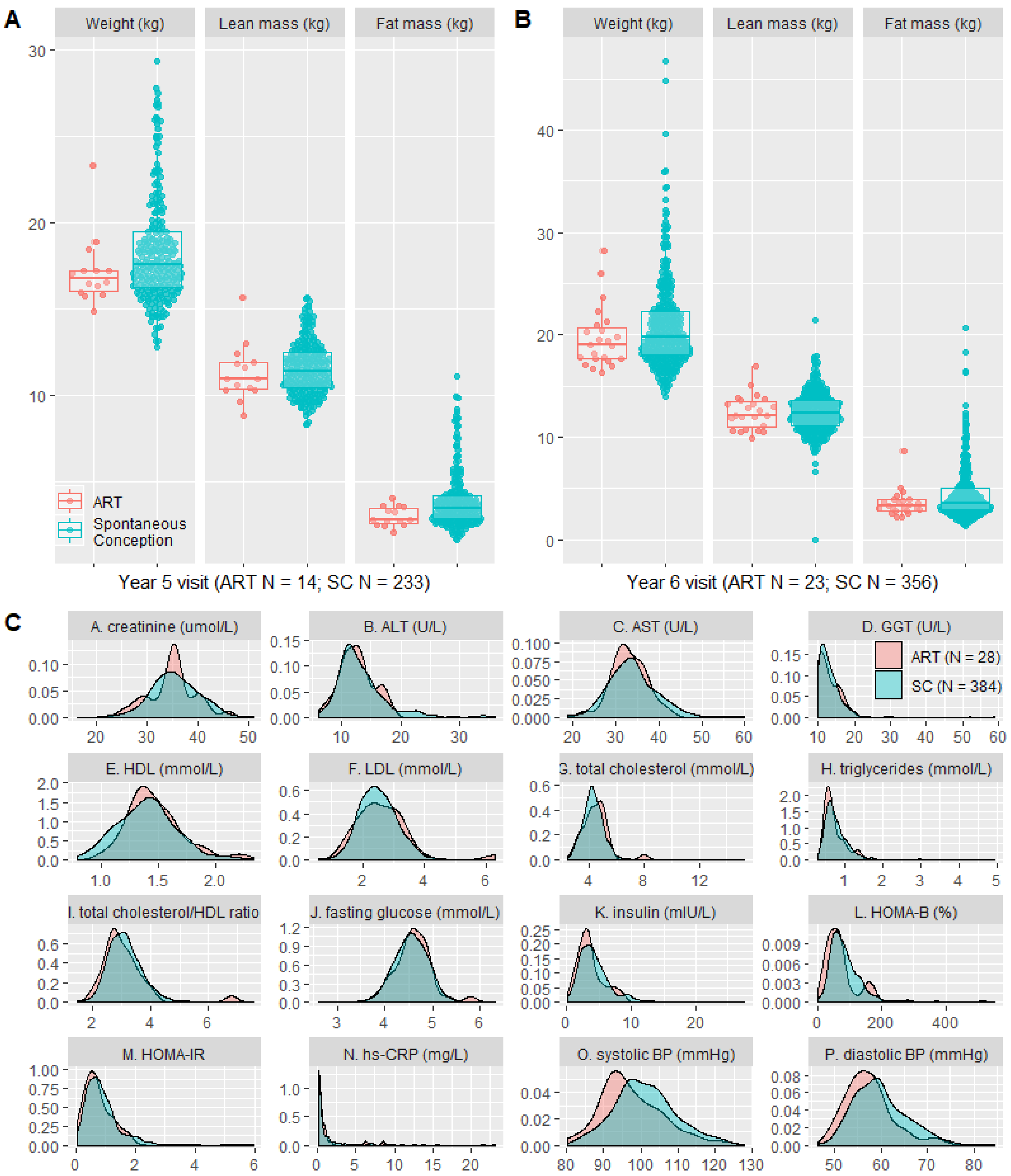

Supplemental Figure 2. **QMR-measured body composition at years 5 (A) and 6 (B) and distributions of serum cardiometabolic biomarkers and blood pressure at 6 years (C), by ART status**. (A) and (B) show raw distributions and box plot hinges at 75% percentile, median, and 25% percentile with whiskers extending to 1.5 times the interquartile range. (C) show smoothed density plots. Shown with all available measurements. Kg = kilograms; ALT = alanine aminotransferase; AST = aspartate transaminase; GGT = gamma-glutamyl transferase; HDL = high-density lipoprotein; LDL = low-density lipoprotein; HOMA-B = homeostatic model assessment of beta-cell function; HOMA-IR = homeostatic model assessment of insulin resistance; hs-CRP = high-sensitivity C-reactive protein; BP = blood pressure

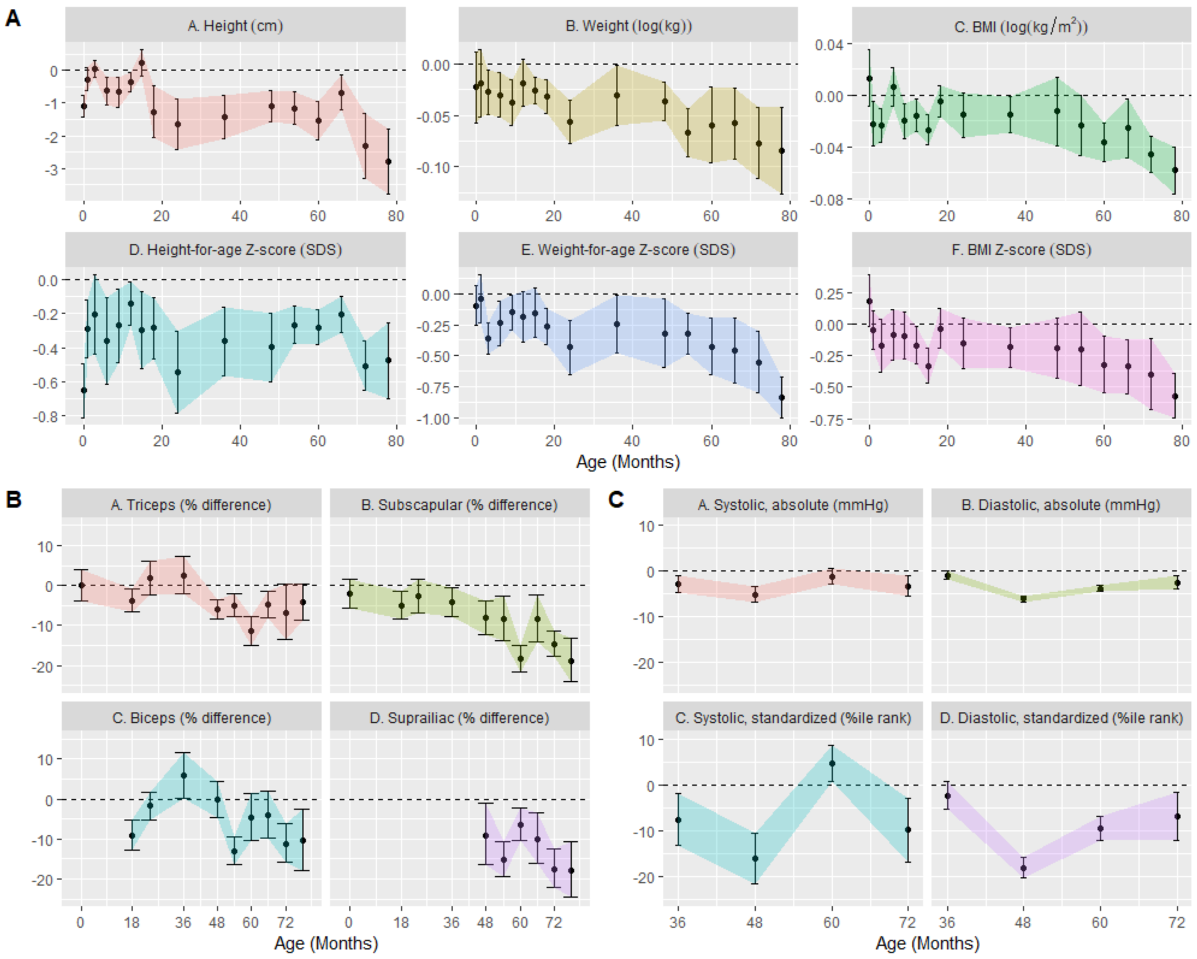

Supplemental Figure 3. **Difference in anthropometrics (A), skinfold thickness (B), and blood pressures (C) comparing ART- and spontaneously-conceived foetuses, estimated by collaborative-targeted maximum likelihood estimation (C-TMLE).** Outcome and treatment models were fit for each outcome and time point allowing for the following to be chosen as covariates: maternal age, education, ethnicity, household income, height, pre-pregnancy BMI, parity, and any tobacco exposure in the home; paternal age, height, weight, diabetes history, and high blood pressure history; child size, and polygenic risk score for adult adiposity. Models were fit via cross-validated, ensemble machine learning (SuperLearner) with a library comprising GLM with interactions, Bayes GLM, observed mean, neural net, and XG Boost as algorithms. Propensity score selection models were 5-fold cross-validated. cm = centimeters; kg = kilograms; m = meters; SDS = standard deviation score; colors only indicate different outcome measures. Samples sizes are identical to the main analyses: (A) 93/83; 83/69; 86/71; 84/68; 80/70; 79/68; 79/67; 72/55; 80/60; 84/64; 84/57; 86/64; 80/61; 80/63; 82/59; 77/59; (B) 91/81; 69/48; 73/54; 82/62; 81/55; 82/63; 75/59; 77/62; 80/55; 75/57; (C) 75/53; 72/44; 70/48; 66/47.

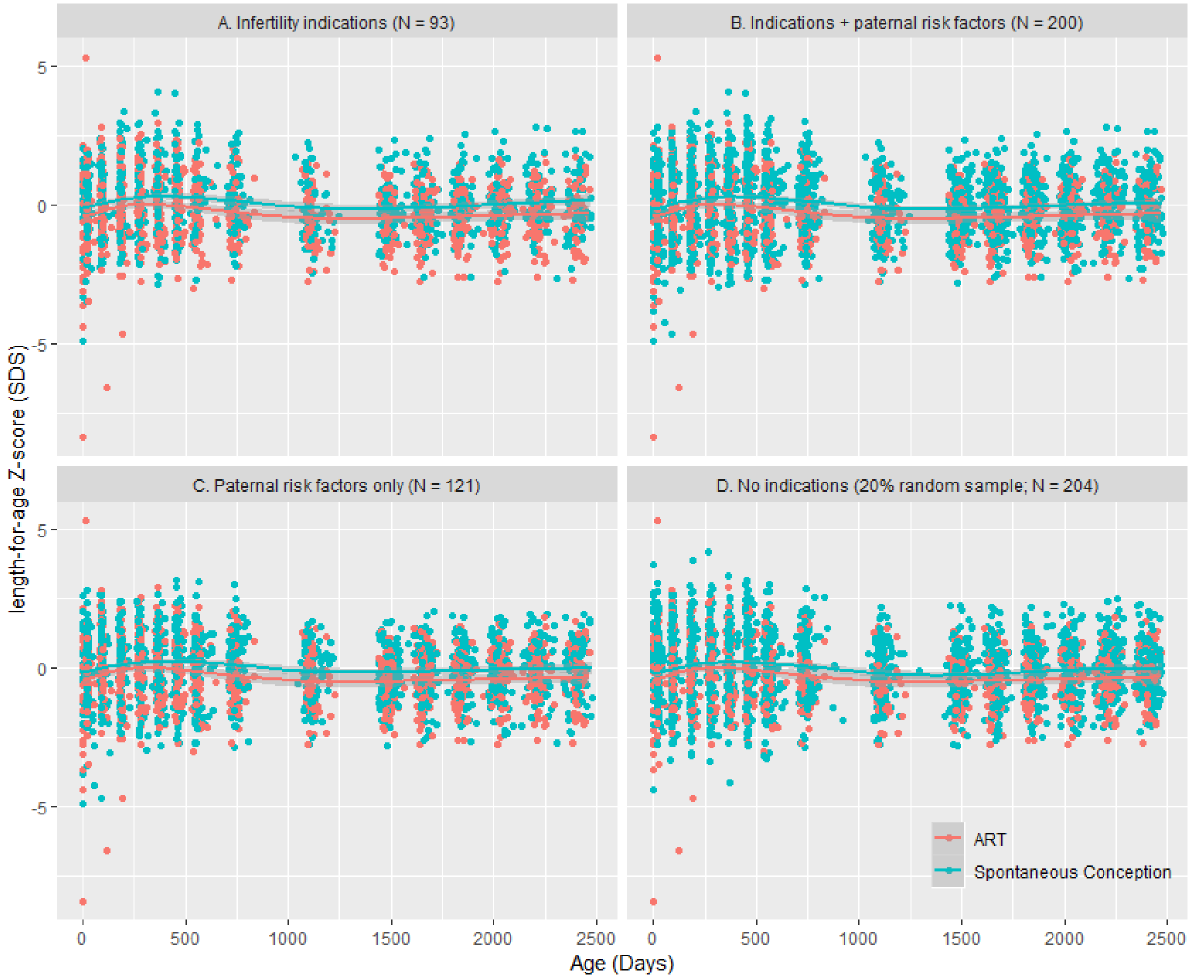

Supplemental Figure 4. **Length-for-age Z-score and ART status over time, by comparison cohort.** Distributions for length/height-for-age Z-score are plotted with fitted smoothed (LOESS) trend lines and shaded 95% confidence interval regions comparing the ART-conceived children (N = 83) against a comparison spontaneously conceived (SC) cohort (sample sizes given in the headers). Plot (A) shows the comparison against our original selection criteria: history of 2 or more past miscarriages; medications with potential fertility indications (*e.g.* aspirin, hormones, thyroid, weight loss); history of PCOS, endometriosis, ovarian cysts, fibroids, or thyroid disorders (hyper- or hypo-). Plot (B) uses the same criteria as (A) adding paternal risk factors for infertility (age > 40 years; BMI > 35; diabetes mellitus; or high blood pressure). Plot (C) compares against SC with paternal risk factors only. Plot (D) compares against a randomly drawn cohort of SC with no indications.

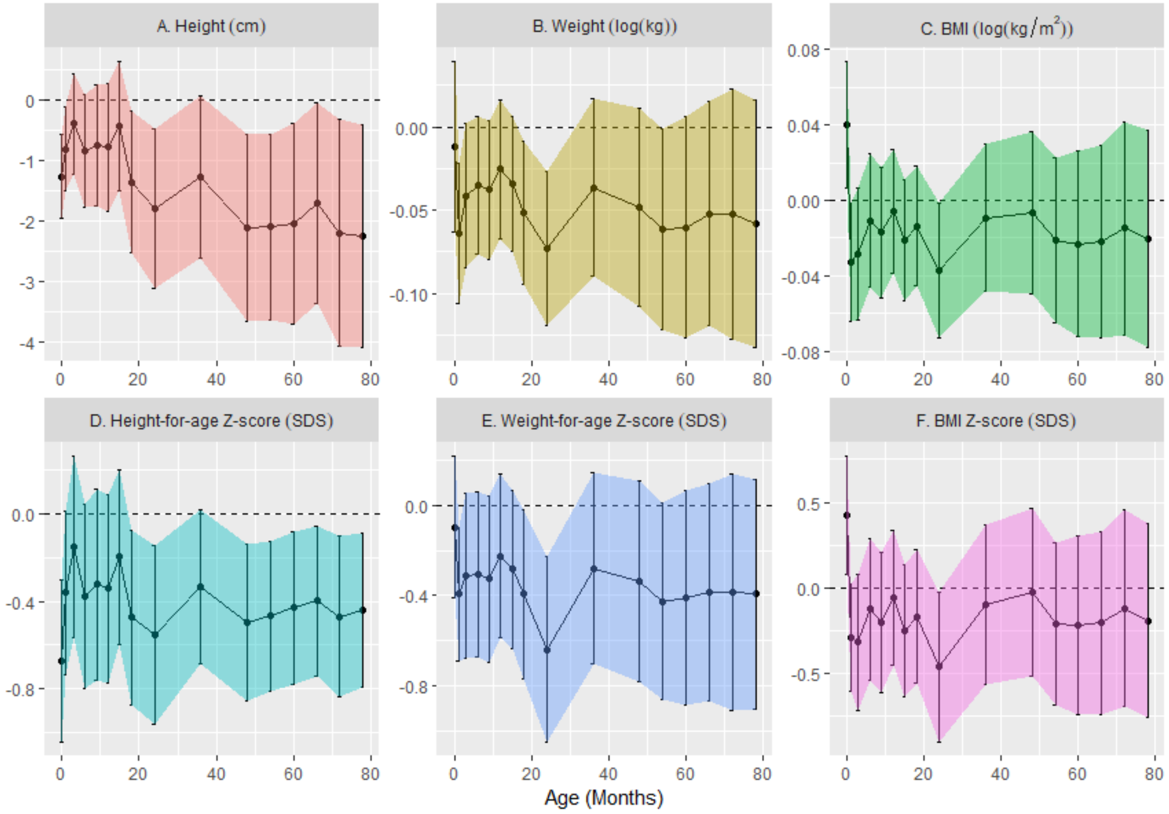

Supplemental Figure 5. **Associations between ART status and anthropometry, accounting for differential follow-up at each study visit.** Point estimates and 95% confidence intervals for multivariable linear regressions adjusted for maternal age, education, ethnicity, household income, height, pre-pregnancy BMI, parity, and any tobacco exposure in the home; paternal height and weight; child size, and polygenic risk score for adult adiposity. Observations were weighted by their inverse probability of exposure and censorship at each visit. Weights were calculated by estimating 17 propensity scores (1 for the probability of treatment and 1 for the probability of censorship at each of 16 visits), taking their inverse (with the inverse probability of exposure stabilized by the exposure prevalence), and then taking the product of the inverse probability of exposure and the corresponding visit-specific censorship weight with no additional adjustments for multiple testing. Missing data were multiple imputed by chained equations to properly account for sampling variability. cm = centimeters; kg = kilograms; m = meters; SDS = standard deviation score; colors only indicate different outcome measures.

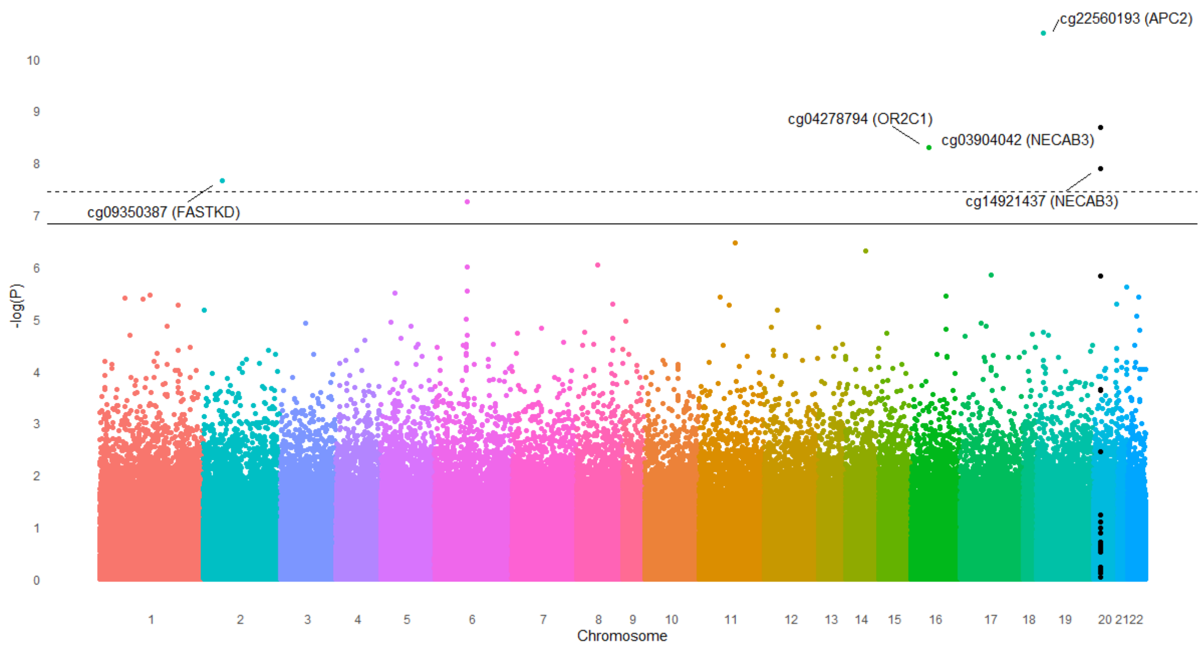

Supplemental Figure 6. **EWAS of ART status**. CpGs significant at epigenome-wide level ($p < 3.6 \times 10^{-8}$) labelled. Adjusted for maternal ethnicity, age, parity, pre-pregnancy BMI, and child sex. Performed post-hoc at reviewer's request. *NECAB3*-annotating CpGs shown in black with two epigenome-wide significant hits. Three other isolated epigenome-wide hits (cg09350387, cg04278794, cg22560193) were not part of the *a priori* candidate sites and not followed up as part of this study.

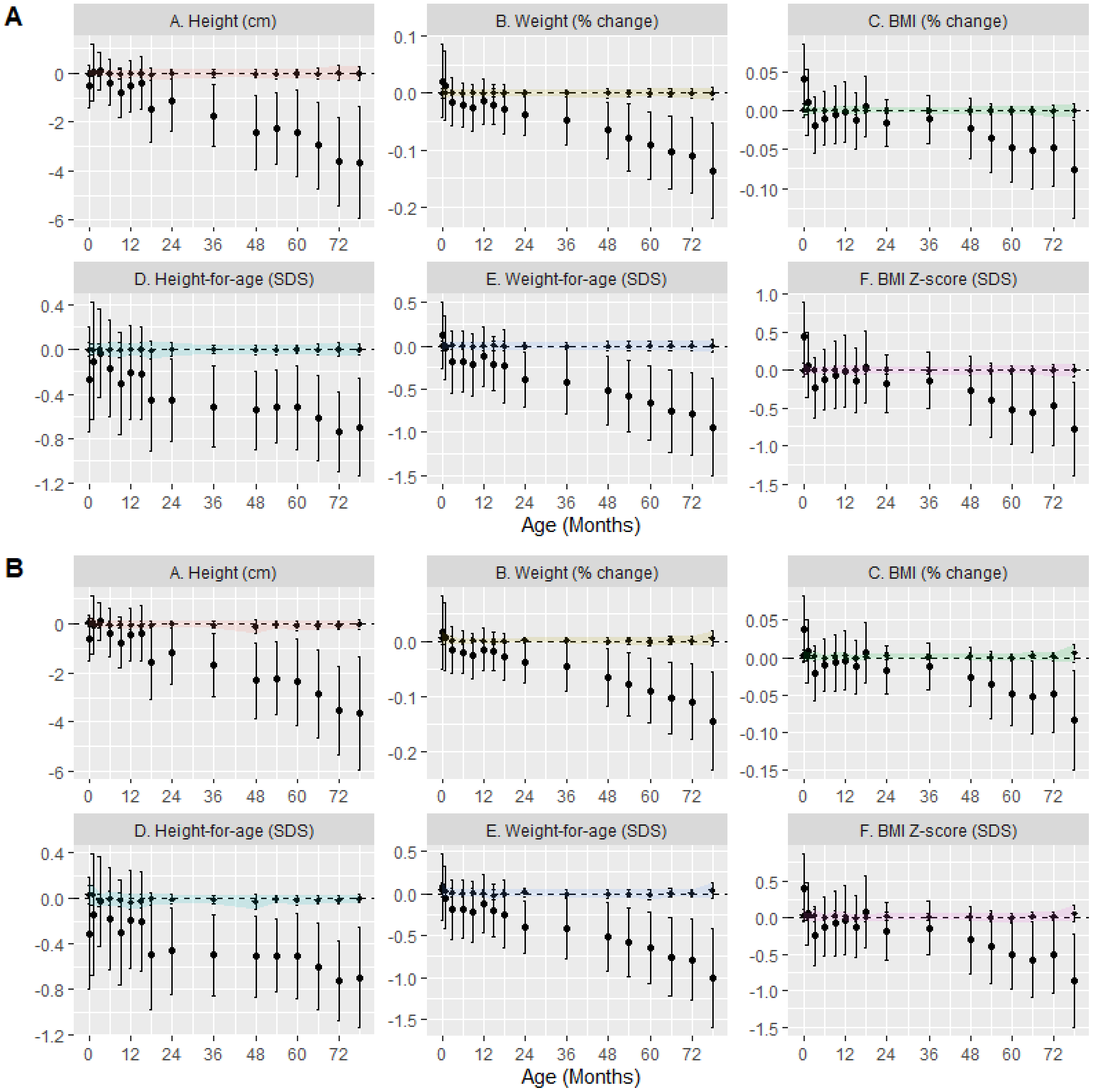

Supplemental Figure 7. **Negative control analyses of mediation by cg03904042 (A) and cg27146050 (B) methylation in maternal mid-pregnancy peripheral blood, target trial subcohort**. The solid lines show the estimates and Wald-type 95% confidence intervals for the hypothetical average direct treatment effect of assigning all subjects to ART (versus spontaneous conception) while holding maternal methylation at the untreated (SC) level. Point estimates and standard errors were estimated by parametric g-computation in 100 bootstrapped datasets. Covariates and approach were identical to the fetal cord tissue methylation mediation simulations amongst the subfertile subcohort (target trial). Shaded regions represent mediation by top *NECAB3*-annotating hit (cg03904042) and top HIF3A-annotating hit (cg27146050) in panels (A) and (B), respectively. Samples sizes were identical to Figures 3a and 6 (SC/ART): (a) 93/83; 83/69; 86/71; 84/68; 80/70; 79/68; 79/67; 72/55; 80/60; 84/64; 84/57; 86/64; 80/61; 80/63; 82/59; 77/59.

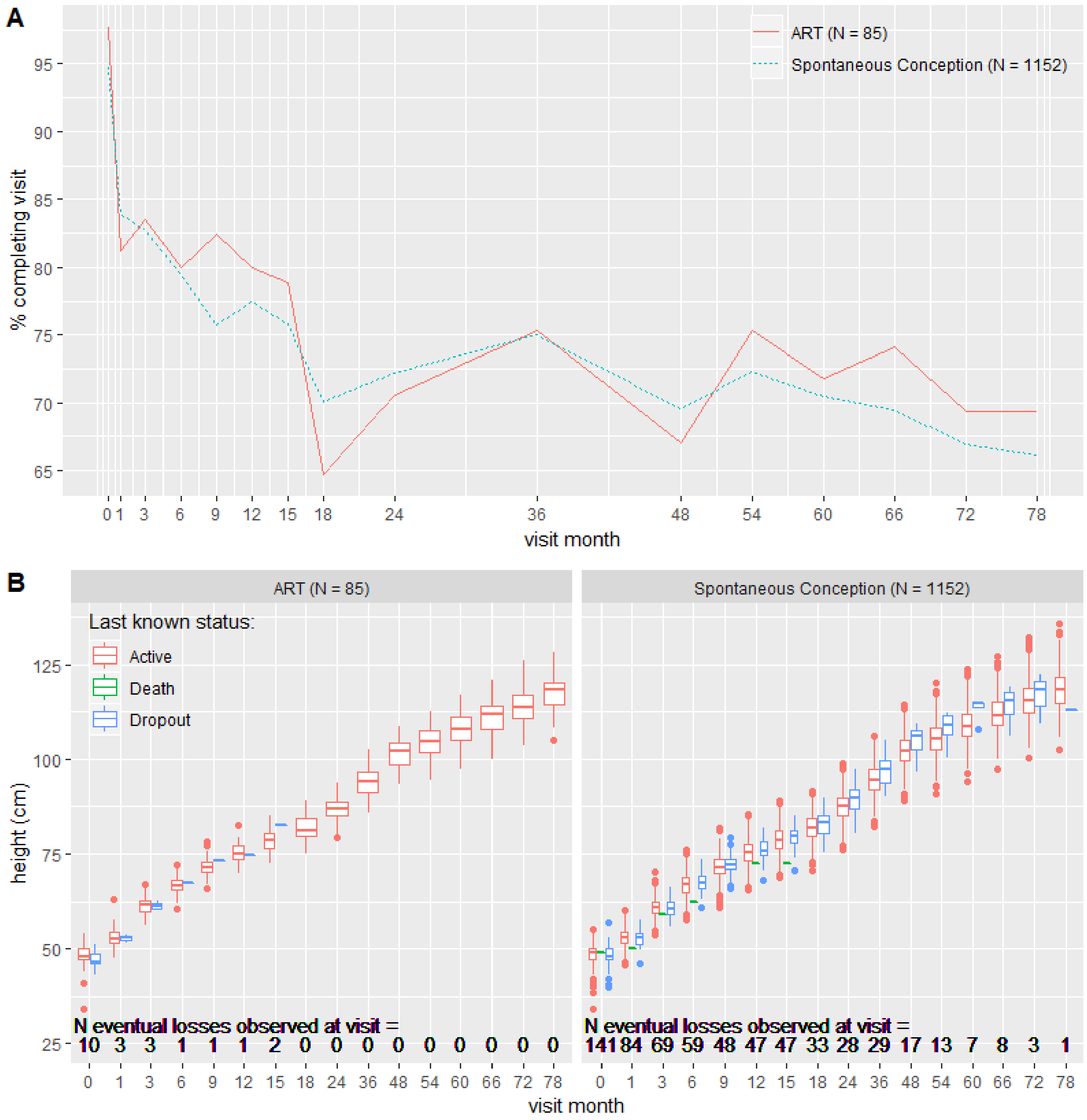

Supplemental Figure 8. **Subject retention (A) and distribution of observed heights (B), by ART and eventual loss-to-follow-up status**. Retention patterns do no differ greatly by ART status (A). Among ART, those who are eventually lost to follow-up are shorter or similar to those retained (B). Among the spontaneously conceived those eventually lost are either the same or taller (B). Box plots hinges at 75% percentile, median, and 25% percentile with whiskers extending to 1.5 times the interquartile range.

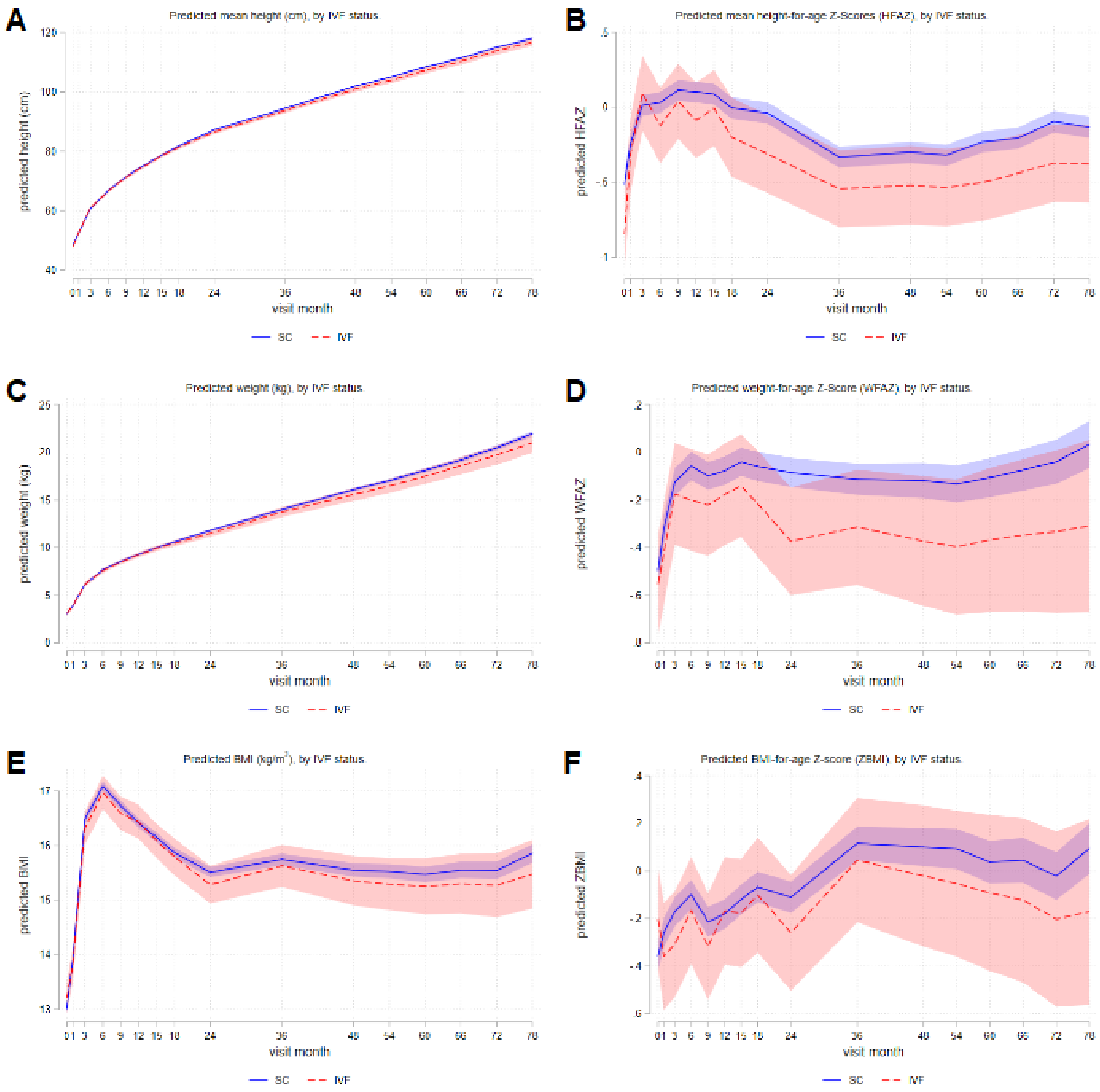

Supplemental Figure 9. **Predicted average anthropometrics by ART status, under a linear mixed effects model with individual-level random intercepts and slopes.** (A) predicted mean height (B) predicted mean height-for-age (C) predicted mean weight (D) predicted mean weight for age (E) predicted mean BMI and (F) predicted mean BMI-for-age are all lower among IVF offspring (with varying degrees of precision) consistent with other findings. Shaded regions represent 95% confidence intervals for the predicted mean anthropometric measure.

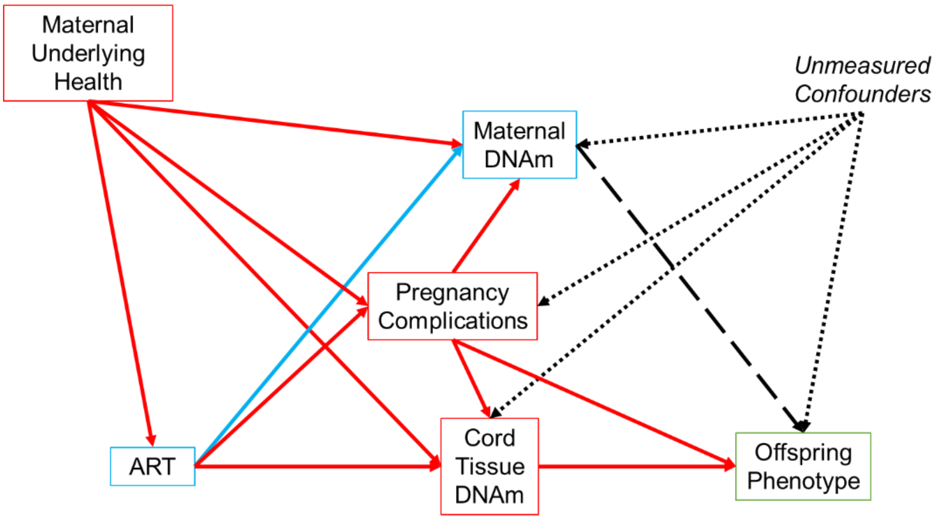

Supplemental Figure 10. **Negative Control Mediation Model by Maternal DNA methylation (DNAm)**. This diagram illustrates the causal relationships between ART, maternal and fetal epigenome, and offspring phenotype implied by a fetal genomic imprinting hypotheses. Notably, if maternal underlying heath, ART status, and pregnancy complications are appropriately controlled for (indicated by red lines), there should be no direct effect of Maternal DNAm on offspring phenotype, and accordingly the proportion mediated by Maternal CpGs should be zero (as indicated by an absence of a solid blue path from ART through Maternal DNAm to Offspring Phenotype). If a mediated effect of Maternal DNAm is non-zero this indicates either (a) the presence of unmeasured confounders (dotted edges) or (b) a direct effect of maternal DNAm (dashed edge).
