# Supplementary material for: Analyses of child cardiometabolic phenotype following assisted reproductive technologies using a pragmatic trial emulation approach
Source: Nat Commun. 2021 Sep 23;12:5613. doi: 10.1038/s41467-021-25899-4 (PMC8460697; doi:10.1038/s41467-021-25899-4)
Supplement: Supplementary file 3 — Description of Additional Supplementary Material [file 41467_2021_25899_MOESM3_ESM.docx]

**Supplemental Data Legend:**

**Supplemental Data 1**: “Candidate CpGs (N = 281) arranged by p-value of association with ART status in descending order with associated references”
