# Supplementary material for: Analyses of child cardiometabolic phenotype following assisted reproductive technologies using a pragmatic trial emulation approach
Source: Nat Commun. 2021 Sep 23;12:5613. doi: 10.1038/s41467-021-25899-4 (PMC8460697; doi:10.1038/s41467-021-25899-4)
Supplement: Supplementary file 5 — Reporting Summary [file 41467_2021_25899_MOESM5_ESM.pdf]

nature research

Corresponding author(s): Jonathan Huang

Last updated by author(s): Apr 5, 2021

Reporting Summary

Nature Research wishes to improve the reproducibility of the work that we publish. This form provides structure for consistency and transparency in reporting. For further information on Nature Research policies, see our Editorial Policies and the Editorial Policy Checklist.

Statistics

For all statistical analyses, confirm that the following items are present in the figure legend, table legend, main text, or Methods section.

n/a	Confirmed	
☐	☒	The exact sample size (*n*) for each experimental group/condition, given as a discrete number and unit of measurement
☐	☒	A statement on whether measurements were taken from distinct samples or whether the same sample was measured repeatedly
☐	☒	The statistical test(s) used AND whether they are one- or two-sided<br>*Only common tests should be described solely by name; describe more complex techniques in the Methods section.*
☐	☒	A description of all covariates tested
☐	☒	A description of any assumptions or corrections, such as tests of normality and adjustment for multiple comparisons
☐	☒	A full description of the statistical parameters including central tendency (e.g. means) or other basic estimates (e.g. regression coefficient) AND variation (e.g. standard deviation) or associated estimates of uncertainty (e.g. confidence intervals)
☐	☒	For null hypothesis testing, the test statistic (e.g. *F*, *t*, *r*) with confidence intervals, effect sizes, degrees of freedom and *P* value noted<br>*Give P values as exact values whenever suitable.*
☒	☐	For Bayesian analysis, information on the choice of priors and Markov chain Monte Carlo settings
☐	☒	For hierarchical and complex designs, identification of the appropriate level for tests and full reporting of outcomes
☐	☒	Estimates of effect sizes (e.g. Cohen's *d*, Pearson's *r*), indicating how they were calculated

Our web collection on statistics for biologists contains articles on many of the points above.

Software and code

Policy information about availability of computer code

Data collection	Genomic data were called using GenomeStudio Genotyping Module v1.8, Methylation Module v1.8, and GenCall (Illumina, San Diego); polygenic risk scores were constructed using PLINK 1.9; questionnaire, anthropometric, MRI, and other non-genomic data were stored in a LORIS (Longitudinal Online Research and Imaging System) database
Data analysis	All data processing, analyses, and visualizations for this study were conducted in either Stata 15.1 SE (StataCorp, College Station, Texas) using base and gformula macros or RStudio 1.2.1335 (RStudio, Inc., Boston, Massachusetts) / R 3.6.0 (R Core Team, Vienna Austria) using the stats 3.6.0, sva 3.22, minfi 1.22.1, haven 2.3.1, tidyverse 1.3.1, ggplot2 3.3.2, readxl 1.3.1, ggpubr 0.4.0, ctmle 0.1.2, and SuperLearner 2.0.26 packages as well as their respective dependencies. Custom code and sample data to reproduce analyses can be found at: github.com/jhuang35/ivf_growth/ or doi:10.5281/zenodo.4662336

For manuscripts utilizing custom algorithms or software that are central to the research but not yet described in published literature, software must be made available to editors and reviewers. We strongly encourage code deposition in a community repository (e.g. GitHub). See the Nature Research guidelines for submitting code & software for further information.

Data

Policy information about availability of data

All manuscripts must include a data availability statement. This statement should provide the following information, where applicable:

- Accession codes, unique identifiers, or web links for publicly available datasets
- A list of figures that have associated raw data
- A description of any restrictions on data availability

Data are available upon reasonable request to the GUSTO study executive committee. A working overview of available data and variables can be found at: sicsdatavault.sg/gusto/. The corresponding author may be contacted for further details. Methylome data are deposited with the NCBI Gene Expression Omnibus

(http://www.ncbi.nlm.nih.gov/geo/) under Series accession number GSE158064. Other data are not currently publicly available due to applicable institutional and governmental regulations. Output data to reproduce figures can be found: github.com/jhuang35/ivf_growth/ or doi:10.5281/zenodo.4662336

Field-specific reporting

Please select the one below that is the best fit for your research. If you are not sure, read the appropriate sections before making your selection.

☒ Life sciences ☐ Behavioural & social sciences ☐ Ecological, evolutionary & environmental sciences

For a reference copy of the document with all sections, see nature.com/documents/nr-reporting-summary-flat.pdf

Life sciences study design

All studies must disclose on these points even when the disclosure is negative.

Sample size	All eligible singleton pregnancies in our representative prospective cohort were included for analyses.
Data exclusions	Twin pregnancies were excluded a priori from analyses due to differences in pregnancy course and physiology from singletons.
Replication	This was a human observational study, no experiments were performed. We investigated 187 candidate DNA methylation CpGs drawn from past studies. Our findings in fetal cord tissue replicate 1 previous site each from Sharp, et al. Hum Mol Genet. 2017, and Novakovic, et al. Nat Commun. 2019. Reasons for non-replication of other sites include differences in target tissue, phenotypes, sequencing platforms, and sample sizes.
Randomization	Randomization to expectant management of infertility is not feasible. We follow best practices for non-randomized studies of interventions by emulating a target trial; as well as extensive covariate adjustment and propensity score weighting; machine learning for flexible covariate control; and full-conditional specification multiple imputation for missing covariate information.
Blinding	This cohort study was not recruited on the basis of treatment status, thus data collection was not conducted differentially on this basis. As a secondary data analyses, it was not possible to blind the analysis, however exhaustive sensitivity analyses were conducted to identify source of bias and explain observed differences

Reporting for specific materials, systems and methods

We require information from authors about some types of materials, experimental systems and methods used in many studies. Here, indicate whether each material, system or method listed is relevant to your study. If you are not sure if a list item applies to your research, read the appropriate section before selecting a response.

Materials & experimental systems

n/a	Involved in the study	
☒	☐	Antibodies
☒	☐	Eukaryotic cell lines
☒	☐	Palaeontology and archaeology
☒	☐	Animals and other organisms
☐	☒	Human research participants
☒	☐	Clinical data
☒	☐	Dual use research of concern

Methods

n/a	Involved in the study	
☒	☐	ChIP-seq
☒	☐	Flow cytometry
☒	☐	MRI-based neuroimaging

Human research participants

Policy information about studies involving human research participants

Population characteristics	This study was conducted within the "Growing Up in Singapore Towards healthy Outcomes" (GUSTO) prospective birth cohort as described in (Soh, et al. 2014). Briefly, 1247 women were recruited between June 2009 and September 2010 from women attending first trimester ultrasound data scans at the two major public maternity units in Singapore: National University Hospital (NUH) and KK Women's and Children's Hospital (KKH). Women were eligible if 18 years and older, Singaporean citizens or permanent residents, with self-reported homogenous ethnic ancestry (Chinese, Indian, Malay), intended to deliver at the either of the recruitment hospitals and reside in Singapore for the next 5 years. Women greater than 14 weeks of gestation, receiving chemotherapy, psychotropic medications, or having an existing type I diabetes mellitus diagnosis at the time of recruitment were excluded. Women who ultimately did not agree to donate birth tissues (cord, placenta, cord blood) were also excluded.
Recruitment	Recruitment occurred at early pregnancy visit and were generally representative of the source population of pregnancy women. The major reasons women were screened out or refused were unlikely to bias the study and were in fact strengths: intention to deliver at other hospitals (27%), more than 14 weeks gestation (24%), and non-homogenous ancestry (19%). Since recruitment occurred in early pregnancy, the major potential selection biases for the purposes of this study would have

	been failure-to-conceive, pregnancy loss, and subsequent loss-to-follow up. We address this extensively through inverse-probability of selection (censorship) weighted models and multiple imputation of missing data
Ethics oversight	Study protocols following the principles of the Declaration of Helsinki and were approved by the respective ethics committees for the two hospitals: National Healthcare Group Domain Specific Review Board (NUH) and SingHealth Centralized Institutional Review Board (KKH).

Note that full information on the approval of the study protocol must also be provided in the manuscript.
